# Metabolic Profile of Calcium Oxalate Stone Patients with Enteric Hyperoxaluria and Impact of Dietary Intervention

**DOI:** 10.3390/nu16162688

**Published:** 2024-08-13

**Authors:** Roswitha Siener, Charlotte Ernsten, Thomas Welchowski, Albrecht Hesse

**Affiliations:** 1 University Stone Center, Department of Urology, University Hospital Bonn, 53127 Bonn, Germany; s4cherns@uni-bonn.de (C.E.); albrecht-hesse@web.de (A.H.); 2 Department of Medical Biometry, Informatics and Epidemiology, Medical Faculty, University of Bonn, 53127 Bonn, Germany; thomas.welchowski@ukbonn.de

**Keywords:** urolithiasis, kidney stones, Crohn’s disease, secondary hyperoxaluria, intestinal oxalate absorption, calcium, oxalate, bowel resection, fat malabsorption, diet

## Abstract

This study investigated the risk profile and the impact of dietary intervention in calcium oxalate stone formers with enteric hyperoxaluria under controlled, standardized conditions. Thirty-seven patients were included in the study. Dietary and 24-h urinary parameters were obtained on the self-selected diet and a balanced, standardized diet. Tests for [^13^C_2_]oxalate absorption, calcium- and ammonium chloride-loading were performed. Mean [^13^C_2_]oxalate absorption was 18.8%. A significant positive association was observed between urinary oxalate excretion and intestinal oxalate absorption. In addition, urinary oxalate excretion was significantly correlated with dietary oxalate intake. Mean urinary oxalate excretion decreased from 0.841 mmol/24 h on the usual diet to 0.662 mmol/24 h on the balanced diet, corresponding to a reduction of 21.3%. Besides hyperoxaluria, hypocitraturia and hypomagnesuria were the most common urinary abnormalities at baseline, being present in 83.8% and 81.1% of patients, respectively. Urinary citrate increased by 50.9% and magnesium excretion increased by 25.2% on the balanced diet. As a result, the relative supersaturation of calcium oxalate declined significantly (by 36.2%) on the balanced diet. Since 41% of patients on the balanced diet still had a urine volume of less than 2.0 L/24 h, efforts should be made to increase urine volume by increasing fluid intake and reducing intestinal fluid losses. Dietary intervention proved to be effective in reducing urinary oxalate excretion and should be a cornerstone of the treatment of patients with enteric hyperoxaluria.

## 1. Introduction

Enteric hyperoxaluria is a metabolic disorder attributed to an underlying intestinal condition usually associated with fat malabsorption, such as Crohn’s disease with ileal resection, exocrine pancreatic insufficiency, short bowel syndrome, or malabsorptive bariatric surgery [1,2,3]. Enteric hyperoxaluria can result from increased absorption of dietary oxalate secondary to malabsorption of fatty acids or increased oxalate permeability of the intestine. Unabsorbed free fatty acids can bind dietary calcium in the gut lumen, reducing the availability of luminal calcium for complexation with oxalate and increasing the concentration of soluble oxalate available for absorption in the intestine [4,5]. Delivery of unabsorbed fatty acids and bile salts to the colon, the critical site of oxalate absorption in enteric hyperoxaluria, may also increase the permeability of the colonic mucosa, thereby facilitating intestinal oxalate absorption [6,7,8]. In a retrospective population-based cohort study of 297 patients with enteric hyperoxaluria, urolithiasis occurred in 27.3% during a median follow-up of 4.9 years [9]. Hyperoxaluria, an excessive urinary excretion of oxalate, not only can lead to urinary stone formation, but also carries the risk of oxalate nephropathy, chronic kidney disease, and even renal failure [10,11,12,13]. In a study of 68 patients with oxalate nephropathy due to enteric hyperoxaluria, 56% of patients developed end-stage kidney disease during a median follow-up of 31 months [14].

Besides hyperoxaluria, hypocitraturia, a low rate of urinary citrate excretion, is a common urinary risk factor for calcium oxalate stone formation in patients with fat malabsorption [2,15,16]. Urinary citrate is a potent inhibitor of calcium oxalate crystallization and functions primarily by forming a pH-dependent complex with calcium, thereby increasing the solubility of calcium and reducing the concentration of free calcium in the urine [17,18]. The major physiological determinant of proximal-tubule reabsorption and urinary excretion of citrate are changes in acid-base homeostasis [17,19]. In patients with fat malabsorption, hypocitraturia appears to be mainly due to a loss of bicarbonate in the stool, which results in a metabolic acid load [20,21]. At lower intracellular and/or luminal pH values, citrate reabsorption in the renal proximal tubule is increased, resulting in a decrease in urinary citrate excretion [22]. In addition, urinary magnesium excretion was found to be significantly lower among patients with Crohn’s disease and urolithiasis compared to healthy subjects [3].

Current pharmacological treatment options for calcium oxalate stone formers with enteric hyperoxaluria are limited to oral calcium and magnesium supplementation and alkali therapy [23,24]. Although dietary modification is recommended, the impact of dietary intervention on urinary risk factors for kidney stone formation associated with fat malabsorption is still unclear. In particular, there is a paucity of knowledge regarding the dietary habits of calcium oxalate stone patients with enteric hyperoxaluria, and findings on the metabolic risk profile of this patient population under controlled, standardized conditions are limited. The objective of the present study was to characterize the clinical, metabolic, and nutritional risk factors for stone formation associated with enteric hyperoxaluria and to determine the effect of dietary intervention on the urinary risk profile for calcium oxalate nephrolithiasis.

## 2. Materials and Methods

### 2.1. Patients

Calcium oxalate stone patients with a history of hyperoxaluria secondary to a previously diagnosed enteric disease associated with intestinal malabsorption, i.e., Crohn’s disease, exocrine pancreatic insufficiency, small bowel resection, or bariatric surgery, were enrolled in the study. Eligible patients included adult women and men with a documented calculus from a recent stone event containing at least 50% calcium oxalate. Stone analysis was performed using Fourier transform infrared spectroscopy (FTIR) (PerkinElmer, Waltham, MA, USA). Hyperoxaluria, defined as baseline urinary oxalate excretion ≥ 0.450 mmol/24 h [25], had to have been diagnosed prior to the study, when the patients were on their usual diets. Medications and supplements that could modify urinary risk factors for stone formation, such as thiazide diuretics, allopurinol, citrate, and pyridoxine, were discontinued at least 4 weeks before and during the study. Patients did not receive any nutritional counseling and were reminded to maintain their usual diet prior to enrollment. Patients were referred to the University Stone Center of the Department of Urology at the University Hospital Bonn for inpatient metabolic evaluation according to a controlled, standardized program. The study was approved by the Ethics Committee of the Medical Faculty of the University of Bonn (approval number 430/19), and informed consent was obtained from each patient.

### 2.2. Study Design

Anthropometric, clinical, 24-h urinary parameters, medical history, and a 7-day dietary protocol were obtained from the stone formers while they were consuming their usual diets. Trained staff provided detailed instructions for recording dietary intake. The types and amounts of all foods consumed were described in detail by the patients. The composition of the diet was calculated using the PRODI 5.3 computer program (Nutri-Science GmbH, Freiburg, Germany). The oxalate content of all foods and beverages measured in our laboratory was entered into the software database [26,27,28]. Sodium intake was determined by 24-h urinary sodium excretion [29]. In the subsequent phase, the patients were fed a balanced, standardized diet for a period of eleven days [23,29]. Water intake from beverages was 2.5 L per day. All of the foods and meals prepared for the diet were accurately weighed. Participants were required to consume their allotted meals. Fluid intake was monitored by urine volume. Compliance was supervised by trained personnel. Participants collected 24-h urine samples during their usual diet and on the balanced, standardized diet. Analysis of 24-h urinary parameters was performed as previously described [30]. Ion-activity-product indices of calcium oxalate and uric acid were determined [23,31]. Relative supersaturation of calcium oxalate and uric acid was calculated using the iterative computer program EQUIL2 [32]. Glomerular filtration rate (eGFR) was estimated using the CKD-EPI study equation for adults [33]. External laboratory quality certificates were available for urinary-stone analysis and each urine parameter. The calcium-loading test was performed on days 2 and 3, the ammonium chloride-loading test on day 4, and the [^13^C_2_]oxalate-absorption test on days 9 and 10 under controlled, standardized conditions [34].

### 2.3. [^13^C_2_]Oxalate Absorption Test

The [^13^C_2_]oxalate-absorption test was used to examine the gastrointestinal oxalate absorption of the patients [35,36]. The test was conducted under controlled, standardized conditions on two consecutive days. The procedure for the first day was designed to achieve a steady state to allow for appropriate calibration. On the second day, patients received 50 mg of sodium [^13^C_2_]oxalate, equivalent to 33.8 mg of [^13^C_2_]oxalic acid, in the morning after an overnight fast, and they collected fractional 24-h urine. Labeled and unlabeled oxalate was quantified by gas chromatography–mass spectrometry. Absorption was expressed as a percentage of the dose of labeled oxalate. Hyperabsorption of oxalate is defined as intestinal absorption of greater than or equal to 10% [23,37].

### 2.4. Ammonium Chloride-Loading Test

The ammonium chloride-loading test was performed to diagnose distal renal tubular acidosis (dRTA) [23,24]. The test was carried out on patients whose urine pH was consistently ≥ 5.8 for several days. The amount of NH_4_Cl administered was 0.1 g/kg body weight. A cut-off urine pH of 5.4 in the day profile was used to identify incomplete dRTA.

### 2.5. Calcium-Loading Test

The calcium-loading test was performed to diagnose the different forms of hypercalciuria [23]. Patients with a urinary calcium excretion of ≥5 mmol/24 h on the habitual diet were considered. Patients began fasting the day before after dinner at 6 p.m., except for 300 mL of distilled water at 8 p.m. and 11 p.m. On the test day, the patients received 600 mL of distilled water at 7 a.m. and 300 mL at 11 a.m. At 9 a.m., 1 g of calcium was administered orally in a total volume of 300 mL together with a standardized breakfast. The first urine sample was obtained between 7 and 9 a.m. in the fasting state (2 h fasting urine). The second urine sample was collected between 9 a.m. and 1 p.m. (4 h urine). Creatinine and calcium were measured in the first and second urine samples. The ratio of calcium (mmol/L) to creatinine (mmol/L) was determined. Absorptive hypercalciuria was defined as a calcium-to-creatinine ratio of ≤0.337 in the first urine sample and ≥0.564 in the second urine sample and renal hypercalciuria as a calcium-to-creatinine ratio of ≥0.338 in the first urine sample and ≥0.564 in the second urine sample.

### 2.6. Statistical Analysis

The study was designed to evaluate a continuous response variable (24-h urinary oxalate excretion) in paired groups. According to previous investigations, a true difference of 0.125 was assumed between post-exposure and pre-exposure measurements of urinary oxalate excretion, with a standard deviation of 0.25. A sample size of 35 subjects is required to reject the null hypothesis that the difference in response is zero with a power of 0.8. The type I error probability is 0.05. The variables were evaluated for normal distribution using the Shapiro–Wilk test. The nonparametric Wilcoxon rank-sum test was used to determine differences in urinary parameters and nutrient intake associated with the self-selected diet and the balanced diet. Categorical variables were compared using the binomial test for symmetry. This corresponds to an exact test version of McNemar’s test. Exceptions were made if the categorical variables had more than two categories. In such cases, pairwise 2 × 2 binomial tests for symmetry were conducted and *p* values were corrected by a false-discovery rate of q = 0.05 [38]. Associations between variables were determined using Spearman’s rank correlation. All statistical tests were two-tailed for exploratory analysis only with a significance level of α = 0.05, excluding the effects of multiple testing. Statistical analyses were performed with IBM SPSS version 27.0 (SPSS Inc., Chicago, IL, USA) and the statistical software R 4.3.1 [39].

## 3. Results

### 3.1. Patients

A total of 37 calcium oxalate stone patients with enteric hyperoxaluria, 10 (27%) females and 27 males (73%) aged 25 to 72 years, were included in the study. Patient characteristics are shown in Table 1. Medians and interquartile ranges for these values are depicted in Table S1. The most frequent underlying enteric conditions were Crohn’s disease (70.3%) and small-bowel resection for other reasons (21.6%). Chronic diarrhea was common in patients with enteric hyperoxaluria (64.9%). Intestinal oxalate hyperabsorption, defined as oxalate absorption of 10% or greater, was detected in 77.4% of patients. At baseline, hypercalciuria occurred in 13.5% of patients, while distal renal tubular acidosis (dRTA) was absent. The majority of patients (70%) had an estimated glomerular filtration rate (eGFR) of at least 60 mL/min/1.73 m^2^, while the remainder (30%) had CKD stage 3 to 4 (eGFR 15.0 to 59.9 mL/min/1.73 m^2^). No significant associations were observed between eGFR and BMI (ρ = −0.073; *p* = 0.671), age at first stone event (ρ = −0.121; *p* = 0.476), duration of stone disease (ρ = −0.252; *p* = 0.132), total number of stone passages (ρ = −0.327; *p* = 0.103), or [^13^C_2_] oxalate absorption (ρ = −0.065; *p* = 0.730).

The mean age of the patients at the time of the first stone event was 35.4 ± 11.8 years, ranging from 6 to 57 years. The calculi were unilateral in 29.7% and bilateral in 70.3% of the patients. All patients but four had undergone active intervention for urolithiasis. Extracorporeal shock wave lithotripsy (ESWL) had been carried out at least once in 67.6% of patients, and 56.8% of patients had undergone at least one ureteroscopy. Urinary stones had passed spontaneously in 75.7% of patients. A positive family history of urolithiasis was observed in 44.1% of patients, with 23.5% having a first-degree relative affected. Of 37 stone patients, 10 (27.0%) had anatomical anomalies, with renal cysts (6 patients) and stenosis (3 patients: 2 ureteropelvic junction obstruction/subpelvic stenosis and 1 distal urethral stenosis) being the most common. Among all 37 patients, 2 women had undergone total nephrectomy. The cause of loss of kidney was obstruction, especially infection (urosepsis), due to nephrolithiasis.

### 3.2. Urine Composition

The 24-h urine composition of calcium oxalate stone patients with enteric hyperoxaluria on their usual diet and on the balanced, standardized diet is depicted in Table 2. Medians and interquartile ranges are shown in Table S2. On the balanced diet, the ion-activity products of calcium oxalate and uric acid decreased by 46.9% and 48.0%, respectively. In addition, the relative supersaturation of calcium oxalate and uric acid was significantly lower on the balanced diet. Urine volume and pH, as well as urinary excretion of magnesium and citrate, increased significantly, while urine density and excretion of sodium, chloride, sulfate, and oxalate declined significantly on the balanced diet. No statistically significant changes were noted in other urinary parameters.

A positive correlation was observed between eGFR and urinary citrate excretion on both the self-selected diet (ρ = 0.612; *p* < 0.001) and the balanced diet (ρ = 0.726; *p* < 0.001). In addition, eGFR correlated significantly and positively with urinary pH (ρ = 0.387; *p* = 0.018), density (ρ = 0.334; *p* = 0.044), and potassium excretion (ρ = 0.385; *p* = 0.018) and correlated significantly and negatively with urinary ammonium excretion (ρ = −0.378; *p* = 0.025).

Apart from hyperoxaluria, the most common urinary abnormality in 24-h urine on the self-selected diet was hypocitraturia, which affected 84% of all calcium oxalate stone formers with enteric hyperoxaluria (Table 3). During the balanced diet, urinary citrate excretion greater than or equal to 1.7 mmol/24 h was observed in 35% of patients. Urine pH below 5.8 was present in 70% of patients on the self-selected diet but in only 43% of patients on the balanced diet. Pairwise comparisons between each urine-pH category and the corresponding complement set indicate differences between groups for ranges of urine pH ≥ 5.8 and the complement set <5.8 (adjusted *p* value 0.019). Hyperoxaluria was less common in patients on the balanced diet. Urinary magnesium excretion below 3.0 mmol/24 h was diagnosed in 81% of patients on the usual diet and was still found in 70% of patients on the balanced diet. Urinary calcium excretion between 5.0 and 7.9 mmol/24 h occurred in 14% of patients on the self-selected diet and in 5% of patients on the balanced diet, while urinary calcium excretion ≥ 8.0 mmol/24 h did not occur in any patient. A urine volume of less than 2 L/24 h was found in 62% of patients on the usual diet and was still found in 41% of patients on the balanced diet.

### 3.3. Nutrient Intake

The nutrient intakes for the self-selected diet and the balanced diet are shown in Table 4. Medians and interquartile ranges are presented in Table S3. The mean daily intakes of energy, protein, methionine, cysteine, fat, saturated fatty acids, monounsaturated fatty acids, cholesterol, sodium, magnesium, total oxalate, soluble oxalate, and alcohol were significantly higher on the self-selected diet, while the mean daily intakes of fiber and polyunsaturated fatty acids were significantly lower.

**Figure 1:**
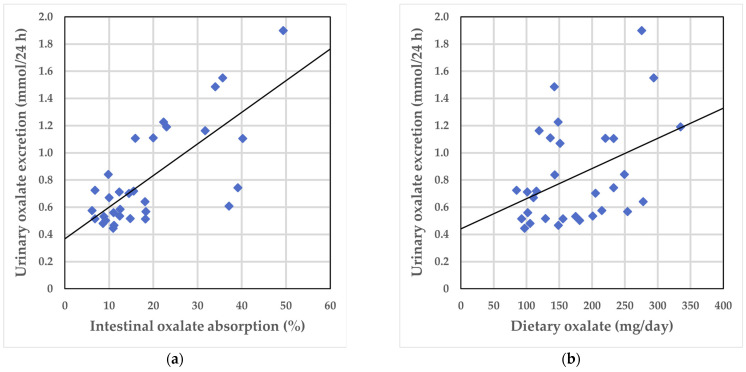
Correlations between urinary oxalate excretion on the self-selected diet and intestinal oxalate absorption and total oxalate intake, respectively: (**a**) Urinary oxalate excretion and intestinal oxalate absorption; (**b**) Urinary oxalate excretion and total oxalate intake. The solid line shows the univariate linear regression.

A significant positive correlation was observed between urinary oxalate excretion on the self-selected diet and intestinal oxalate absorption (ρ = 0.642; *p* < 0.001) (Figure 1a), between urinary oxalate excretion and total oxalate intake (ρ = 0.360; *p* = 0.047) (Figure 1b), and between urinary oxalate excretion and soluble oxalate intake on the self-selected diet (ρ = 0.378; *p* = 0.036).

## 4. Discussion

Enteric hyperoxaluria is associated with an increased risk of urinary stone formation and can lead to CKD and end-stage kidney disease [12]. Identifying and developing treatment options for this patient population is therefore of paramount importance. Oral administration of a novel oxalate-degrading enzyme for 4 weeks only modestly reduced urinary oxalate excretion in patients with enteric hyperoxaluria, and adverse events were relatively common [40]. Although the initial focus should be on dietary therapy, there is a paucity of studies on dietary interventions to reduce urinary oxalate excretion and improve the urinary risk profile in patients with enteric hyperoxaluria. Of the few studies that have evaluated a low-oxalate diet in patients who have undergone ileal resection for various intestinal diseases [4,5] or bariatric surgery [41,42], only patients who had undergone ileal resection showed a reduction in urinary oxalate excretion [4,5]. However, these studies were conducted in relatively small numbers of patients, ranging from four to eleven subjects [4,5,42], with concomitant use of calcium supplements [42], or the results suggest noncompliance with the prescribed diet in patients following bariatric surgery [41].

In the present study, the dietary intervention resulted in a 21.3% reduction in urinary oxalate excretion. Mean urinary oxalate excretion decreased from 0.841 mmol/24 h on the usual diet to 0.662 mmol/24 h on the balanced diet, while total and soluble dietary oxalate intake declined significantly from 175 mg/day and 74 mg/day, respectively, to 121 mg/day and 54 mg/day, respectively. Using the [^13^C_2_]oxalate-absorption test under controlled, standardized conditions, 77.4% of patients were diagnosed with intestinal hyperabsorption of oxalate, defined as oxalate absorption of 10% or more. The mean oxalate absorption in the patients was 18.8%. Intestinal oxalate absorption in patients with enteric hyperoxaluria was similar to that found in a recent study of patients with Crohn’s disease and urolithiasis but approximately twice that of patients with Crohn’s disease without urolithiasis [3]. A positive correlation was observed between urinary oxalate excretion on the usual diet and intestinal oxalate absorption, between urinary oxalate excretion and total oxalate intake, and between urinary oxalate excretion and soluble oxalate intake on the self-selected diet. Although a previous study in patients with marked diarrhea showed a decrease in urinary oxalate excretion after a reduction in dietary fat intake [43], this study found no correlation between dietary fat intake and urinary oxalate excretion. Evaluation of the dietary protocols revealed a high intake of oxalate-rich foods and beverages during the self-selected diet. In particular, high rates of consumption of black, green, and iced tea, as well as of chocolate, rhubarb, nuts, and spinach, was observed. The present findings indicate that enteric hyperoxaluria is attributable to intestinal hyperabsorption of high amounts of dietary oxalate. This study of patients with malabsorptive bowel disease also suggests that even moderate restriction of dietary oxalate intake can result in a considerable reduction in urinary oxalate excretion. Even small changes in urinary oxalate concentration have a strong effect on crystallization, as the urinary solubility product of calcium oxalate is affected ten times more by a decline in urinary oxalate concentration than by an equimolar decrease in calcium concentration [44]. A retrospective study of 297 patients with enteric hyperoxaluria concluded that a 20% decrease in urinary oxalate excretion would be expected to result in a 25% reduction in the annual odds of a future stone event [9]. Therefore, the initial management of enteric hyperoxaluria should involve restriction of dietary oxalate intake. A previous study of the outcomes of preventive treatment in stone formers with bowel disease revealed that the lack of effect of an oxalate-lowering intervention may be due to nonadherence to physician advice and/or limited patient knowledge of dietary measures [45]. It is suggested that the use of professional dietitian support could enhance patient education and adherence.

Both magnesium and calcium have the ability to bind oxalate in the intestine, thereby reducing the absorption and urinary excretion of oxalate [46,47]. A recent study in patients with Crohn’s disease and healthy controls revealed a significantly lower urinary excretion of calcium and magnesium in patients with Crohn’s disease under standardized dietary conditions [3]. In the present study, urinary calcium excretion was typically low on both the self-selected and the balanced diets, while the mean dietary calcium on both diets met the recommended intake [23,24]. Accordingly, hypercalciuria was rather rare, occurring in 13.5% of the patients on the self-selected diet and in only 5.4% of the patients on the balanced diet. Furthermore, hypomagnesuria was identified in 81% of patients on the usual diet and was still found in 70% of patients on the balanced diet. Despite a significantly lower dietary magnesium intake on the balanced diet, mean urinary magnesium excretion was 25% higher than on the usual diet. It is hypothesized that the high oxalate intake on the self-selected diet resulted in a reduction in the bioavailability of dietary magnesium for intestinal absorption, as was observed in a previous study [48]. It is recommended that magnesium supplementation and, in patients with low urinary calcium excretion, calcium supplementation be considered as treatment options for patients with enteric hyperoxaluria [3,23,24]. No statistically significant changes were observed in urinary excretion of calcium, potassium, ammonium, phosphate, and uric acid, which may be due to similar dietary intakes of calcium, potassium, phosphorus, and purines on the self-selected diet and the balanced diet.

Low urine volume is a significant risk factor for calcium oxalate stone formation, contributing to an elevated relative supersaturation of calcium oxalate [49]. In the present study, despite comparable water intake on both dietary regimens, urine volume showed a significant rise during the balanced diet. Diarrhea and malabsorption have been reported to be the main intestinal alterations associated with alcohol consumption [50]. High dietary fat intake could also exacerbate diarrhea and lead to increased fecal fluid loss [5,13,43]. Therefore, it is postulated that the reduction and modification of dietary fat intake and the avoidance of alcohol consumption may have contributed to the observed decrease in urine volume. In general, measures should be taken to increase urine volume to at least 2.0 L/24 h by increasing fluid intake and reducing intestinal fluid losses where possible.

Hypocitraturia is a common urinary abnormality in patients with malabsorptive intestinal conditions [2,51]. Urinary citrate, an important inhibitor of crystallization, is a recognized protective factor against calcium oxalate stone formation, primarily because citrate forms soluble complexes with urinary calcium, thereby reducing supersaturation and precipitation of calcium oxalate [17,18]. In the present study, hypocitraturia was diagnosed in 84% of patients on the usual diet. Mean urinary citrate excretion exhibited a notable 51% increase, from 0.949 mmol/24 h on the self-selected diet to 1.432 mmol/24 h on the balanced diet. The major determinant of urinary citrate excretion is an alteration in acid–base homeostasis [17,19,22]. In the present study, the acid–base changes were reflected in a significant increase in urine pH on the balanced diet. A 24-h urine pH of less than 5.8 was observed in 70% of patients on the usual diet, 22% of whom had a urine pH of 5.4 or less, and in 43% of patients on the balanced diet. The proposed mechanism is that changes in systemic pH alter intracellular pH, resulting in changes in intracellular citrate metabolism, which then alter citrate reabsorption and hence urinary citrate excretion [17]. Low urinary pH and citrate excretion in patients with intestinal diseases suggest loss of alkali from chronic diarrhea [52]. It is hypothesized that the restriction of dietary fat intake and cessation of alcohol consumption on the balanced diet and the concomitant improvement in diarrhea and maintenance of alkali equivalents were responsible for the improvement in urinary citrate excretion [43,50]. Since there was no difference in potassium intake between the two diets, it is suggested that the increase in urinary citrate excretion on the balanced diet was due to a reduction in fecal bicarbonate loss rather than a dietary alkali load from fruits and vegetables [21,53]. In addition to dietary changes, alkali therapy is strongly recommended for patients with enteric hyperoxaluria and hypocitraturia.

Hyperoxaluria is not only a risk factor for urinary stone formation but has also been implicated in the pathogenesis of chronic kidney disease [11,12]. Enteric hyperoxaluria has been associated with oxalate nephropathy, defined as the deposition of calcium oxalate crystals within the renal tubulointerstitial compartment with impaired kidney function [54]. Chronic kidney disease (CKD) is a progressive disease that ultimately leads to end-stage renal disease (ESRD) requiring renal-replacement therapy through dialysis or kidney transplantation. In patients with pre-existing CKD, increased urinary oxalate excretion was reported to be independently associated with a higher risk of CKD progression and ESRD [55]. In addition, a longitudinal observational cohort study of individuals without a history of CKD at baseline found that higher urinary oxalate excretion was significantly associated with up to a 35% higher risk of incident CKD and up to a 134% higher risk in patients with a malabsorptive condition [56]. In the present study, CKD stage 3 or 4 was diagnosed in 30% of the patients. Notably, eGFR was found to be significantly correlated with urinary citrate excretion on both the self-selected diet and the balanced diet. It is hypothesized that CKD reduces urinary citrate excretion, probably secondary to a mild chronic metabolic acidosis [22,57]. Restriction of dietary oxalate intake should be the cornerstone of management of enteric hyperoxaluria.

The metabolic evaluation of patients with enteric hyperoxaluria indicated a high risk of calcium oxalate and uric acid stone formation on the usual diet. The ion-activity product of calcium oxalate decreased significantly (by 47%) on the balanced diet, primarily due to the significant reduction in urinary oxalate excretion and the significant increase in urine volume, as well as in magnesium and citrate excretion. The ion-activity product of uric acid decreased significantly and to a similar extent, which can be ascribed to the increase in urine volume and pH. The significant decrease in the relative supersaturation of calcium oxalate and uric acid supports these results. Reducing the risk of uric acid stone formation in patients with enteric hyperoxaluria is relevant because calcium oxalate stones can also occur as a mixture with uric acid [58].

Despite the significant reduction in the risk of stone formation, it must be emphasized that a urine volume of less than 2.0 L/24 h was recorded in 62% of patients on the usual diet and still in 41% of patients on the balanced diet. Therefore, the focus of dietary management in patients with fat malabsorption should also be on urine volume. In patients with enteric hyperoxaluria, fluid intake may need to be increased significantly to compensate for the volume depletion associated with chronic diarrhea. The quantity of fluid required to achieve a urine volume of at least 2.0 to 2.5 L per 24 h should be distributed evenly throughout the day [28]. In the present study, nutritional therapy in the form of a balanced mixed diet proved effective in reducing the risk of stone formation and should be a fundamental part of the treatment of stone patients with malabsorptive conditions. Dietary intervention could potentially have a significant impact on reducing urinary stone formation and protecting against hyperoxaluria-induced kidney disease. Alkali therapy and, depending on whether hypomagnesuria and/or hyperoxaluria is found, oral magnesium and calcium supplementation should be considered as additional treatment options in patients with fat malabsorption.

## 5. Conclusions

Kidney stone patients with enteric hyperoxaluria are at increased risk of developing CKD. The present study suggests that enteric hyperoxaluria is due not only to hyperabsorption of oxalate secondary to various malabsorptive intestinal diseases, but also to increased dietary intake of oxalate. The results of this study demonstrate that dietary intervention has a significant impact on urinary risk factors for calcium oxalate stone formation. The cornerstone of treatment for enteric hyperoxaluria should be restriction of dietary oxalate and avoidance of oxalate-rich foods. In patients with fat malabsorption, the focus of dietary management should also be on urine volume. Future research on dietary measures to limit oxalate intake and/or absorption in patients with enteric hyperoxaluria is needed to evaluate whether reducing urinary oxalate excretion can prevent the development or progression of CKD. To assess the potential long-term effects of dietary changes on clinical outcomes, larger studies of longer duration are warranted. In particular, the effects of diets other than the balanced mixed diet studied, such as a lacto-vegetarian or a Mediterranean diet, on urinary parameters and the risk of calcium oxalate stone formation should be investigated.

## Figures and Tables

**Table 1:** Characteristics of calcium oxalate stone patients with enteric hyperoxaluria.

	Mean ± SD *n* (%)
Number of patients	37
Gender (women/men)	10/27 (27.0/73.0)
Age (years)	48.6 ± 11.4
BMI (kg/m^2^) ^a^	24.2 ± 4.2
BMI < 18.5 kg/m^2^	1/36 (2.8)
BMI 18.5–24.9 kg/m^2^	19/36 (52.8)
BMI 25.0–29.9 kg/m^2^	13/36 (36.1)
BMI ≥ 30 kg/m^2^	3/36 (8.3)
Enteric condition	
Crohn’s disease with bowel resection	26/37 (70.3)
Length of small bowel resection (cm) ^b^	104 ± 47
Small bowel resection	8/37 (21.6)
Ileus	2/8 (25.0)
Mesenteric vein thrombosis	2/8 (25.0)
Volvulus	1/8 (12.5)
Ileal neuroendocrine tumor	1/8 (12.5)
Benign small intestine tumor	1/8 (12.5)
Inflammatory cecal tumor	1/8 (12.5)
Chronic pancreatitis	2/37 (5.4)
Bypass surgery	1/37 (2.7)
Chronic diarrhea	24/37 (64.9)
Hypertension	8/37 (21.6)
Type 2 diabetes	2/37 (5.4)
[^13^C_2_] oxalate absorption (%) ^c^	18.8 ± 11.7
[^13^C_2_] oxalate absorption ≥ 10.0% ^c^	24/31 (77.4)
Distal renal tubular acidosis ^d^	0/30 (0.0)
Hypercalciuria	5/37 (13.5)
Absorptive hypercalciuria	1/5 (20.0)
Renal hypercalciuria	0/5 (0.0)
Idiopathic hypercalciuria	4/5 (80.0)
eGFR (mL/min/1.73 m^2^)	74.7 ± 25.1
eGFR ≥ 90 mL/min/1.73 m^2^	10/37 (27.0)
eGFR 60.0–89.9 mL/min/1.73 m^2^	16/37 (43.2)
eGFR 45.0–59.9 mL/min/1.73 m^2^	6/37 (16.2)
eGFR 30.0–44.9 mL/min/1.73 m^2^	3/37 (8.1)
eGFR 15.0–29.9 mL/min/1.73 m^2^	2/37 (5.4)
Family history of stones ^e^	15/34 (44.1)
Age at first stone event (years)	35.4 ± 11.8
Duration of stone disease (years)	13.2 ± 11.4
Stone passages in the past year ^f^	11.4 ± 13.9
Total number of stones passages ^g^	43.9 ± 62.0
Laterality	
Bilateral	26/37 (70.3)
Right	5/37 (13.5)
Left	6/37 (16.2)
Anatomical anomalies	10/37 (27.0)
Nephrectomy	1/37 (2.7)
Nephrectomy and kidney cysts	1/37 (2.7)
Kidney cysts	5/37 (13.5)
Stenosis	3/37 (8.1)
Type of stone removal	
Spontaneous passage	28/37 (75.7)
Extracorporeal shock wave lithotripsy	25/37 (67.6)
Ureteroscopy	21/37 (56.8)
Percutaneous nephrolithotomy	10/37 (27.0)
Open surgery	4/37 (10.8)

Abbreviations: BMI, body mass index; eGFR, estimated glomerular filtration rate; SD, standard deviation. ^a^ *n* = 36 (10 women, 26 men) due to missing data. ^b^ *n* = 11 (3 women, 8 men) due to missing data. ^c^ *n* = 31 (9 women, 22 men) due to missing data. ^d^ *n* = 30 (7 women, 23 men) due to missing data. ^e^ *n* = 34 (10 women, 24 men) due to missing data. ^f^ *n* = 27 (9 women, 18 men) due to missing data. ^g^ *n* = 26 (9 women, 17 men) due to missing data.

**Table 2:** Urine parameters on the self-selected diet and the balanced diet.

	Self-Selected Diet *n* = 37 Mean ± SD	Balanced Diet *n* = 37 Mean ± SD	*p* Value
Volume (L/24 h)	1.866 ± 0.796	2.254 ± 0.636	0.032
Density (g/cm^3^)	1.010 ± 0.004	1.006 ± 0.003	<0.001
Urinary pH	5.70 ± 0.46	5.87 ± 0.50	0.036
Sodium (mmol/24 h)	159 ± 68	116 ± 52	0.004
Potassium (mmol/24 h)	46 ± 20	48 ± 20	0.571
Calcium (mmol/24 h)	2.92 ± 1.70	2.67 ± 1.40	0.172
Magnesium (mmol/24 h)	2.14 ± 1.39	2.68 ± 1.50	<0.001
Ammonium (mmol/24 h) ^a^	42.7 ± 21.0	35.5 ± 14.9	0.070
Chloride (mmol/24 h)	185 ± 80	126 ± 51	<0.001
Phosphate (mmol/24 h)	27.4 ± 7.3	25.0 ± 5.1	0.077
Sulfate (mmol/24 h)	16.4 ± 5.8	13.8 ± 4.4	0.018
Creatinine (mmol/24 h)	13.26 ± 3.07	12.74 ± 3.22	0.063
Uric acid (mmol/24 h)	2.87 ± 0.94	2.65 ± 0.83	0.083
Oxalate (mmol/24 h)	0.841 ± 0.392	0.662 ± 0.294	<0.001
Citrate (mmol/24 h)	0.949 ± 1.489	1.432 ± 1.421	0.011
RS Uric acid	2.368 ± 2.162	1.291 ± 1.126	<0.001
RS Calcium oxalate	9.254 ± 3.819	5.907 ± 3.504	<0.001
AP Uric acid index (10^−9^)	1.702 ± 1.722	0.885 ± 0.844	<0.001
AP Calcium oxalate index	2.313 ± 1.055	1.229 ± 0.789	<0.001

Abbreviations: AP, activity product; RS, relative supersaturation; SD, standard deviation. ^a^ *n* = 35 (10 women, 25 men) due to missing data.

**Table 3:** Urinary abnormalities on the self-selected diet and the balanced diet.

	Reference Range	Self-Selected Diet *n* = 37 *n* (%)	Balanced Diet *n* = 37 *n* (%)	*p* Value
Volume (L)	<2.000	23 (62.2)	15 (40.5)	0.096
	≥2.000	14 (37.8)	22 (59.5)	
Urine pH	<5.40	8 (21.6)	7 (18.9)	1.000
	5.40–5.79	18 (48.6)	9 (24.3)	0.074
	≥5.80	11 (29.7)	21 (56.8)	0.019
Calcium (mmol/24 h)	<5.0	32 (86.5)	35 (94.6)	0.375
	5.0–7.9	5 (13.5)	2 (5.4)	
	≥8.0	0 (0.0)	0 (0.0)	-
Magnesium (mmol/24 h)	<3.0	30 (81.1)	26 (70.3)	0.219
	≥3.0	7 (18.9)	11 (29.7)	
Uric acid (mmol/24 h)	<4.0	32 (86.5)	34 (91.9)	0.687
	≥4.0	5 (13.5)	3 (8.1)	
Oxalate (mmol/24 h)	<0.45	0 (0.0)	10 (27.0)	0.002
	≥0.45	37 (100.0)	27 (73.0)	
Citrate (mmol/24 h)	<1.7	31 (83.8)	24 (64.9)	0.016
	≥1.7	6 (16.2)	13 (35.1)	

**Table 4:** Nutrient intake on the self-selected diet and the balanced diet.

	Self-Selected Diet *n* = 31 ^a^ Mean ± SD	Balanced Diet *n* = 31 ^a^ Mean	*p* Value
Energy (kcal/day)	2671 ± 718	2355	0.025
Protein (g/day)	99 ± 29	71	<0.001
Methionine (mg/day)	2089 ± 667	1415	<0.001
Cysteine (mg/day)	1306 ± 374	835	<0.001
Fat (g/day)	104 ± 38	81	0.004
SFA (g/day)	46 ± 19	30	<0.001
MUFA (g/day)	37 ± 14	26	<0.001
PUFA (g/day)	13 ± 5	19	<0.001
Cholesterol (mg/day)	417 ± 167	195	<0.001
Carbohydrates (g/day)	304 ± 76	327	0.050
Fiber (g/day)	24 ± 9	31	<0.001
Purines (mg/day)	492 ± 193	449	0.337
Sodium (mg/day) ^b^	3801 ± 1562	2300	<0.001
Potassium (mg/day)	3351 ± 944	3390	0.854
Calcium (mg/day)	980 ± 350	977	0.664
Magnesium (mg/day)	434 ± 123	341	<0.001
Phosphorus (mg/day)	1511 ± 446	1432	0.650
Total oxalate (mg/day)	175 ± 68	121	<0.001
Soluble oxalate (mg/day)	74 ± 37	54	0.016
Alcohol (g/day)	13.1 ± 18.2	0	<0.001
Water (mL/day)	3162 ± 839	3437	0.087

Abbreviations: MUFA, monounsaturated fatty acids; PUFA, polyunsaturated fatty acids; SD, standard deviation; SFA, saturated fatty acids. ^a^ *n* = 31 (8 women, 23 men) due to missing data. ^b^ calculated from urinary sodium excretion.

## Data Availability

The data presented in the study are available upon reasonable personal request due to data privacy reasons.

## Supplementary Materials

The following supporting information can be downloaded at: https://www.mdpi.com/article/10.3390/nu16162688/s1, Table S1: Characteristics of calcium oxalate stone patients with enteric hyperoxaluria (Shapiro-Wilk test, median, interquartile range); Table S2: Urine parameters on the self-selected diet and the balanced diet (Shapiro-Wilk test, median, interquartile range); Table S3: Nutrient intakes on the self-selected diet (Shapiro-Wilk test, median, interquartile range).
