# Supplementary material for: Metabolic Profile of Calcium Oxalate Stone Patients with Enteric Hyperoxaluria and Impact of Dietary Intervention
Source: Nutrients. 2024 Aug 13;16(16):2688. doi: 10.3390/nu16162688 (PMC11357492; doi:10.3390/nu16162688)
Supplement: Supplementary file 1 [file nutrients-16-02688-s001.zip › nutrients-3095649-supplementary.pdf]

Table S1. Characteristics of calcium oxalate stone patients with enteric hyperoxaluria (Shapiro-Wilk test, median, interquartile range).

	**Median (IQR)**	***p* Value** [a]
Age (years)	48 (20)	0.694
BMI (kg/m^2) [b]	24.6 (6.9)	0.166
Length of small bowel resection in patients with Crohn's disease and bowel resection (cm) [c]	100 (77)	0.804
[$^{13}C_2$] oxalate absorption (%) [d]	14.8 (13.0)	<0.001
eGFR (mL/min/1.73 m^2)	75.7 (39.9)	0.200
Age at first stone event (years)	39 (18)	0.459
Duration of stone disease (years)	9 (14)	<0.001
Stone passages in the past year [e]	4 (28)	<0.001
Total number of stones passages [f]	20 (45)	<0.001

Abbreviations: BMI, body mass index; eGFR, estimated glomerular filtration rate; IQR, interquartile range. [a] *p*-value: Shapiro-Wilk test. [b] n = 36 (10 women, 26 men) due to missing data. [c] n = 11 (3 women, 8 men) due to missing data. [d] n = 31 (9 women, 22 men) due to missing data. [e] n = 27 (9 women, 18 men) due to missing data. [f] n = 26 (9 women, 17 men) due to missing data.

Table S2. Urine parameters on the self-selected diet and the balanced diet (Shapiro-Wilk test, median, interquartile range).

	**Self-selected diet n = 37 Median (IQR)**	***p* Value [a]**	**Balanced diet n = 37 Median (IQR)**	***p* Value [a]**
Volume (L/24 h)	1.680 (1.020)	0.047	2.060 (0.878)	0.114
Density (g/cm^3)	1.010 (0.006)	0.077	1.006 (0.004)	0.003
Urinary pH	5.68 (0.46)	0.082	5.84 (0.66)	0.244
Sodium (mmol/24 h)	150 (113)	0.659	113 (62)	<0.001
Potassium (mmol/24 h)	45 (22)	0.003	45 (30)	0.071
Calcium (mmol/24 h)	2.68 (2.75)	0.030	2.63 (1.92)	0.184
Magnesium (mmol/24 h)	1.81 (1.31)	<0.001	2.28 (1.67)	0.020
Ammonium (mmol/24 h) [b]	42.2 (27.3)	0.022	32.3 (18.8)	0.132
Chloride (mmol/24 h)	181 (130)	0.775	129 (70)	0.133
Phosphate (mmol/24 h)	27.1 (10.8)	0.760	24.8 (7.5)	0.515
Sulfate (mmol/24 h)	16.9 (9.1)	0.119	14.5 (5.9)	0.862
Creatinine (mmol/24 h)	13.52 (4.85)	0.258	13.41 (5.05)	0.239
Uric acid (mmol/24 h)	2.79 (1.19)	0.510	2.49 (1.07)	0.169
Oxalate (mmol/24 h)	0.702 (0.575)	<0.001	0.574 (0.365)	<0.001
Citrate (mmol/24 h)	0.343 (0.964)	<0.001	0.857 (2.301)	<0.001
RS Uric acid	1.700 (1.974)	<0.001	1.034 (1.179)	<0.001
RS Calcium oxalate	9.574 (5.397)	0.686	5.281 (3.730)	0.002
AP Uric acid index (10^{-9})	1.165 (1.457)	<0.001	0.667 (0.854)	<0.001
AP Calcium oxalate index	2.201 (1.478)	0.550	1.040 (0.961)	<0.001

Abbreviations: AP, activity product; IQR, interquartile range; RS, relative supersaturation. [a] *p*-value: Shapiro-Wilk test. [b] n = 35 (10 women, 25 men) due to missing data.

Table S3. Nutrient intakes on the self-selected diet (Shapiro-Wilk test, median, interquartile range).

	**Self-selected diet n = 31 [a] Median (IQR)**	***p* Value [b]**
Energy (kcal/day)	2548 (1001)	0.588
Protein (g/day)	97 (42)	0.843
Methionine (mg/day)	2027 (771)	0.411
Cysteine (mg/day)	1270 (597)	0.478
Fat (g/day)	92 (61)	0.469
SFA (g/day)	42 (27)	0.385
MUFA (g/day)	34 (24)	0.270
PUFA (g/day)	12 (7)	0.092
Cholesterol (mg/day)	376 (294)	0.225
Carbohydrates (g/day)	293 (82)	0.017
Fiber (g/day)	21.9 (9.6)	0.027
Purines (mg/day)	449 (340)	0.227
Sodium (mg/day) [c]	3452 (2519)	0.542
Potassium (mg/day)	3275 (1795)	0.292
Calcium (mg/day)	890 (345)	0.094
Magnesium (mg/day)	417 (208)	0.399
Phosphorus (mg/day)	1427 (656)	0.055
Total oxalate (mg/day)	151 (118)	0.053
Soluble oxalate (mg/day)	68 (40)	0.037
Alcohol (g/day)	4.5 (20.0)	<0.001
Water (mL/day)	3130 (1268)	0.601

Abbreviations: IQR, interquartile range; MUFA, monounsaturated fatty acids; PUFA, polyunsaturated fatty acids; SFA, saturated fatty acids. [a] n = 31 (8 women, 23 men) due to missing data. [b] *p*-value: Shapiro-Wilk test. [c] calculated from urinary sodium excretion.
